# Analytical and clinical validation of PATHWAY Anti-HER-2/neu (4B5) antibody to assess HER2-low status for trastuzumab deruxtecan treatment in breast cancer

**DOI:** 10.1007/s00428-023-03671-x

**Published:** 2023-10-20

**Authors:** Charo Garrido, Melissa Manoogian, Dhiraj Ghambire, Shawn Lucas, Maha Karnoub, Matthew T. Olson, David G. Hicks, Gary Tozbikian, Aleix Prat, Naoto T. Ueno, Shanu Modi, Wenqin Feng, Judith Pugh, Ching Hsu, Junji Tsurutani, David Cameron, Nadia Harbeck, Qijun Fang, Shirin Khambata-Ford, Xuemin Liu, Landon J. Inge, Patrik Vitazka

**Affiliations:** 1 Daiichi Sankyo, Inc, Basking Ridge, NJ USA; 2 Roche Tissue Diagnostics, Tucson, AZ USA; 3 The University of Rochester Medical Center, Rochester, USA; 4 The Ohio State University Wexner Medical Center, Columbus, OH USA; 5 Department of Medical Oncology, IDIBAPS, University of Barcelona, Barcelona, Spain; 6 University of Hawaiʻi Cancer Center, Honolulu, HI USA; 7 Memorial Sloan Kettering Cancer Center, New York, NY USA; 8 The Innovative Center of Translational Research and Clinical Science for Cancer Therapy, Showa University Hospital, Tokyo, Japan; 9 University of Edinburgh Cancer Centre, Institute of Genetics and Cancer, Edinburgh, UK; 10 Breast Center, Depart of OB&GYN and CCC Munich, LMU University Hospital, Munich, Germany; 11 Teva Pharmaceuticals, Parsippany, NJ USA

**Keywords:** Breast cancer, HER2-low, Companion diagnostic, 4B5

## Abstract

In DESTINY-Breast04 (DB-04), safety and efficacy of HER2-targeted antibody-drug conjugate (ADC) trastuzumab deruxtecan (T-DXd) in previously treated HER2-low unresectable/metastatic breast cancer were established. This manuscript describes the analytical validation of PATHWAY Anti-HER2/neu (4B5) Rabbit Monoclonal Primary Antibody (PATHWAY HER2 (4B5)) to assess HER2-low status and its clinical performance in DB-04. Preanalytical processing and tissue staining parameters were evaluated to determine their impact on HER2 scoring. The recommended antibody staining procedure provided the optimal tumor staining, and deviations in cell conditioning and/or antibody incubation times resulted in unacceptable negative control staining and/or HER2-low status changes. Comparisons between antibody lots, kit lots, instruments, and day-to-day runs showed overall percent agreements (OPAs) exceeding 97.9%. Inter-laboratory reproducibility showed OPAs of ≥97.4% for all study endpoints. PATHWAY HER2 (4B5) was utilized in DB-04 for patient selection using 1340 tumor samples (59.0% metastatic, 40.7% primary, (0.3% missing data); 74.3% biopsy, 25.7% resection/excisions). Overall, 77.6% (823/1060) of samples were HER2-low by both central and local testing, with the level of concordance differing by sample region of origin and collection date. In DB-04, the efficacy of T-DXd over chemotherapy of physician’s choice was consistent, regardless of the characteristics of the sample used (primary or metastatic, archival, or newly collected, biopsy or excision/resection). These results demonstrate that PATHWAY HER2 (4B5) is precise and reproducible for scoring HER2-low status and can be used with multiple breast cancer sample types for reliably identifying patients whose tumors have HER2-low expression and are likely to derive clinical benefit from T-DXd.

**Supplementary Information:**

The online version contains supplementary material available at 10.1007/s00428-023-03671-x.

## Introduction

Targeting of HER2 with blocking antibodies has significantly improved outcomes for patients with high levels of HER2 expression in their tumors, defined as those with gene amplification and/or high protein expression by immunohistochemistry (IHC) [1, 2]. Based on these data, the American Society of Clinical Oncology-College of American Pathologists (ASCO/CAP) codified guidelines for HER2 testing in 2007 [3] and has since updated these guidelines in 2013, 2018, and most recently in 2023 [4–6].

Prior to the most recent update, the 2018 guidelines indicated IHC for HER2 as the primary diagnostic test and recommended reflex testing by in situ hybridization (ISH) to evaluate HER2 gene amplification in cases with an equivocal IHC result [5]. Based on these guidelines, HER2 IHC testing categorized samples into 4 scores: 0, 1+, 2+, and 3+. A score of 3+ is considered HER2-positive and a score of 2+ is equivocal, requiring the sample be reflexed to ISH testing to determine if HER2 gene amplification is present, to identify patients who are suitable candidates for first-generation HER2-targeted therapies. Historically, both IHC 0 and IHC 1+ have been considered HER2-negative. The ASCO/CAP 2023 update retains the same guidance for IHC categorization as the 2018 guidelines, although they now state the clinical relevance of HER2-low [6].

Approximately 40–50% of breast cancer patients have tumors with low HER2 expression (i.e., tumors scoring 1+ or 2+ by IHC and negative by ISH). Initial reports suggested that patients with low HER2-expressing tumors may be distinct from those with IHC 0 tumors in terms of prognosis and response to chemotherapy; however, more recent reports have indicated that any observed differences in prognosis between patients with HER2 IHC 0 and HER2-low tumors are most likely due to different hormone receptor status [7–9]. Historically, these patients have been considered having HER2-negative tumors, with no clinical benefit demonstrable with first-generation HER2-targeted therapies such as trastuzumab and pertuzumab when studied in clinical trials [10, 11].

Trastuzumab deruxtecan (T-DXd), originally named DS-8201a, is an antibody-drug conjugate in which an antibody targeting the HER2 receptor is directly linked with a cytotoxic topoisomerase I inhibitor. In DS8201-A-J101, a first-in-human phase 1 study assessing T-DXd safety and efficacy, T-DXd demonstrated clinically significant anti-tumor activity in patients with HER2-low breast cancer, among other indications [12]. The clinical utility of T-DXd in this population was then demonstrated in DESTINY-Breast04 (DB-04), a randomized, open-label, phase 3 study that selected patients with centrally determined HER2-low (IHC 1+ or IHC 2+ and ISH-negative) metastatic breast cancer who had previously received 1–2 lines of chemotherapy. Prospective selection of patients was via central testing of tumor specimens using the PATHWAY Anti-HER2/neu (4B5) Rabbit Monoclonal Primary Antibody on the VENTANA BenchMark ULTRA staining instrument (the PATHWAY HER2 (4B5) assay) under an investigational use only (IUO) label, with assessment performed by pathologists trained for HER2-low scoring, using the criteria for distinguishing IHC 0 from IHC 1+ set forth in the ASCO/CAP 2018 guidelines, which were the relevant guidelines at the time of this study [5]. In this manuscript, we outline the analytical validation of the PATHWAY HER2 (4B5) assay for HER2-low assessment and scoring, its concordance with prior local HER2 results, and describe the clinical performance of the analytically validated IHC assay by specimen type used in the DB-04 study.

## Materials and methods

### HER2 IHC scoring method

**Fig. 1:**
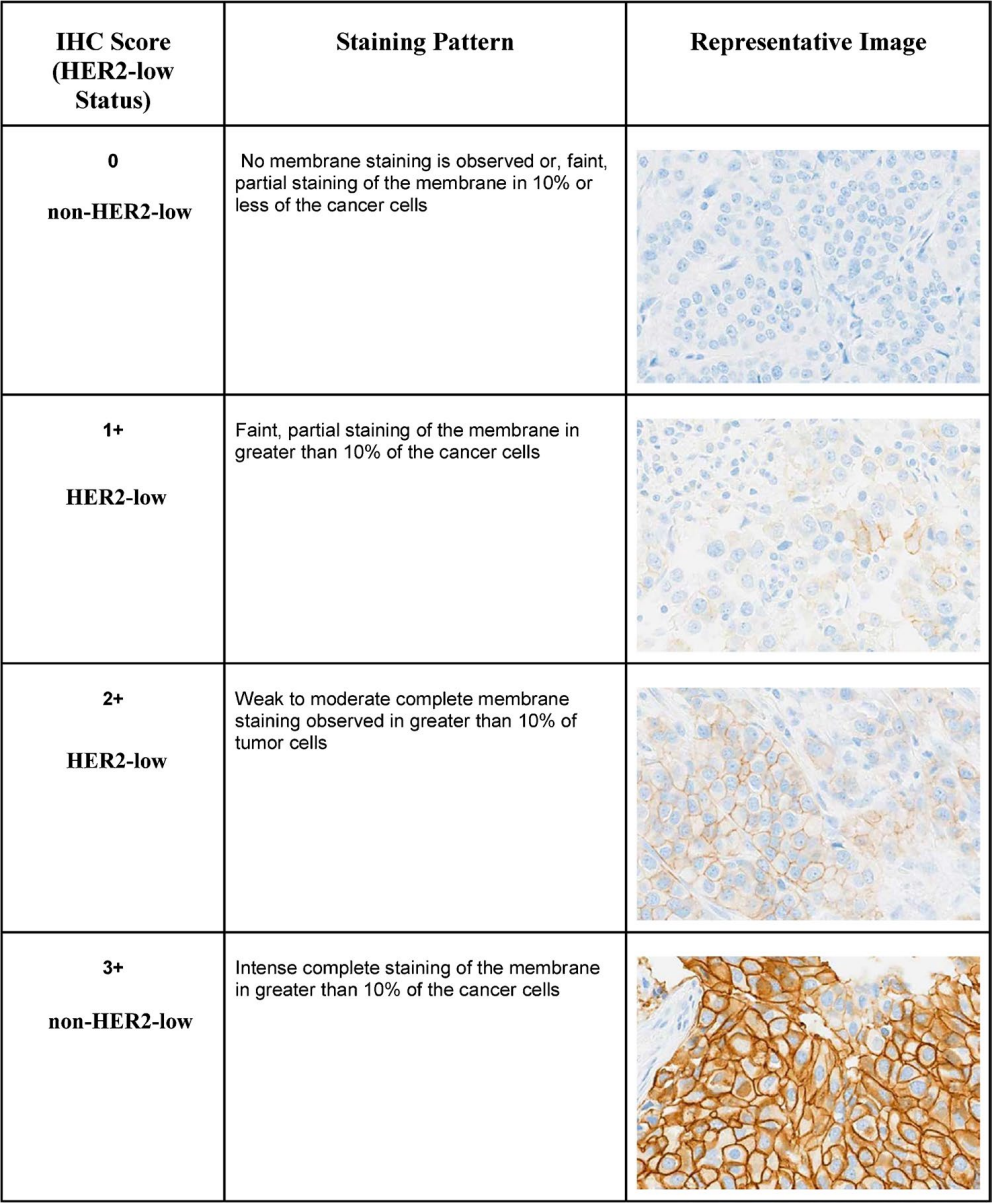
HER2 stain scoring with PATHWAY HER2 (4B5) antibody. HER2, human epidermal growth factor receptor 2; IHC, immunohistochemistry

IHC scoring of HER2 in breast cancer tissues for both the analytical performance and the clinical utility study (study DB-04) were performed according to the 2018 ASCO/CAP guidelines for the assessment of HER2 IHC cut-offs, which form part of the PATHWAY HER2 (4B5) assay instructions for use (IFU) (6). HER2 IHC stained tissues were reviewed by a pathologist and assigned a score (0, 1+, 2+, or 3+) based on current scoring criteria (Fig. 1).

### Tissue specimens and precision and reproducibility studies

Collection of tissue specimens, preanalytical processing studies, immunohistochemistry procedures and assessment of variances in the IHC staining protocol (incubation time of the cell conditioning and antibody incubation) and precision and reproducibility studies are outlined in the Supplementary Methods.

### HER2-low testing in DESTINY-Breast04

DB-04 was a randomized, open-label, phase 3 study for T-DXd versus physician’s choice chemotherapy treatment (randomized 2:1) in patients with HER2-low (IHC 1+ or IHC 2+ and ISH-negative) metastatic breast cancer who had previously received 1–2 lines of chemotherapy [13]. HER2-low status was determined by a central laboratory. Subjects were recruited between December 2018 and December 2021. Samples with a prior available HER2-low result were submitted for central laboratory testing with the IUO-labeled PATHWAY HER2 (4B5) assay, performed on the BenchMark ULTRA staining instrument using the current FDA, CE-marked recommended staining procedure. The most recently available FFPE tumor sample was requested for central testing, regardless of anatomical location or disease stage at the time of collection. If an archival tumor sample was not available, a new tumor collection was required. Subjects with a historical tumor HER2 score of IHC 0 in addition to a HER2-low result were accepted for central screening, but were only included in the study if reclassified as HER2-low by central test (see Supplementary Fig. 1). Scoring of HER2 IHC was performed following the 2018 ASCO/CAP testing guidelines for all IHC cut-offs (Fig. 1). All cases scored as IHC 2+ were assessed for HER2 gene amplification status using the FDA-approved, CE-marked VENTANA INFORM HER2 Dual ISH DNA probe cocktail assay per the manufacturer-recommended staining procedure and evaluated according to the interpretation guide. Pathologists across four different laboratory locations were trained to assess HER2-low status and were qualified to review the clinical trial by completing a final assessment of 40 breast cancer cases. Sample information was collected from clinical sites and entered in the central laboratory database.

To assess concordance between historic (previously known) and central laboratory-assessed HER2-low results, the overall percentage agreement (OPA) and positive percentage agreement (PPA) were calculated. The OPA was calculated as the proportion of total sample results in which historic and central test results agreed. The PPA was calculated as the proportion of historic HER2-low sample results that were also HER2-low by central assessment. The 95% CIs were calculated using the normal approximation of the binomial calculation.

Progression-free survival (PFS) was based on blinded independent central review and defined as the time from the date of randomization to the date of the first radiographic disease progression or death due to any cause, whichever came first. Median PFS was calculated from Kaplan-Meier analysis. The 95% CIs for median PFS were computed using the Brookmeyer-Crowley method. Hazard ratios (HRs) were derived by using the unstratified Cox proportional hazards model, with treatment as the only covariate. Statistical analyses were performed in SAS version 9.4.

## Results

### Evaluation of staining protocol deviations

To determine how deviations from the recommended PATHWAY HER2 (4B5) assay staining procedure affected the assigned HER2 IHC score and HER2-low status, staining involving differing cell conditioning and antibody incubation times were performed at the combinations indicated in Supplementary Table 2. Of the 19 alternative conditions tested (*n*= 3-4 sections per condition), 9 resulted in IHC score changes. Moreover, we observed unacceptable staining for the negative control in 9 conditions. Score changes occurred predominantly with deviations in the cell conditioning times (mostly at 20 and ≥52 min), while increasing antibody incubation times (sometimes by only 8 min) resulted in unacceptable staining of the negative control. Collectively, these data suggest that the current FDA-approved, CE-marked recommended staining procedure should be followed to prevent any impact on HER2 scoring, including HER2-low status assignment.

### Evaluation of preanalytical conditions

The impact of fixative and ischemic time was assessed in studies using the MDA-MB-361 xenograft model system. Delaying fixation for >1 h affected both staining and morphology (data not shown). Choice of fixative was also investigated. As shown in Supplementary Fig. 2, non-formalin-based fixatives altered both the intensity and membrane staining by the PATHWAY HER2 (4B5) assay. For tissues sectioned from 2 µm to 6 µm in thickness, 100% agreement was seen in staining compared with a 4 µm reference specimen, and a decline in agreement was seen upon sectioning at 7 µm (*n* = 12 sections for each slide thickness, Supplementary Table 3). For cut slide stability, repeatability in staining was lost at day 7 for slides stored at high temperature (30 °C±5 °C)/high relative humidity (85%±10%), at month 6 for slides stored at high temperature (30 °C±5 °C)/low relative humidity (15%±10%) or low temperature (5 °C±3 °C)/high relative humidity (85%±10%), and at month 7 for slides stored at low temperature (5 °C±3 °C)/low relative humidity (15%±10%) (*n* = 8–12 sections for each condition, Supplementary Fig. 3).

### Precision and repeatability

**Fig. 2:**
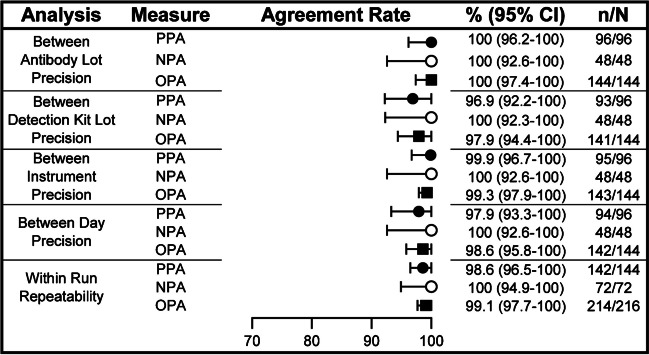
Between antibody lot, detection kit lot, instrument, and day precision and within run repeatability percent agreements for HER2-low or non-HER2-low IHC status. Slides from 24 unique breast carcinoma specimens split evenly among the four scoring types (IHC 0, 1, 2+, 3+), including seven borderline IHC 0/1+ cases. This study involved staining with three antibody lots and three detection kit lots run on three instruments on three different days, with slides stained in duplicate. One reader read all slides. Two-sided 95% CIs were calculated using the percentile bootstrap method from 2000 bootstrap replicates. For observations of 100%, the 95% CIs were calculated using the Wilson score method. CI, confidence interval; HER2, human epidermal growth factor receptor 2; IHC, immunohistochemistry; NPA, negative percent agreement; OPA, overall percent agreement; PPA, positive percent agreement

The agreements for the intermediate precision (between antibody lot, detection kit lot, instrument, and day) and within run repeatability analyses are shown in Fig. 2. Experiments were performed with 24 unique specimens, including 4 borderline for HER2-low. For all parameters, the PPAs and NPAs for determining HER2-low status exceeded 96.9%, and the OPAs for all parameters exceeded 97.9%. Among the parameters tested, the most variability was observed in the comparison between detection kit lots, with a PPA of 96.9% (95% CI 92.2%–100%) and OPA of 97.9% (95% CI 94.4%–100%).

To assess intra- and inter-reader precision in scoring HER2-low status, 100 specimens were read by three internal (Roche) pathologists, and reader agreements were evaluated. For intra-reader precision, the APA and ANA aggregated across all readers was 93.7% (95% CI 90.9%–96.4%) and 92.1% (95% CI 88.0%–95.6%), respectively (Supplementary Table 4). For inter-reader precision, the APA and ANA aggregated across all reader pairs and both reading rounds was 90.4% (95% CI 85.8%–94.3%) and 88.1% (95% CI 82.1%–93.0%) respectively.

### Inter-laboratory reproducibility

**Fig. 3:**
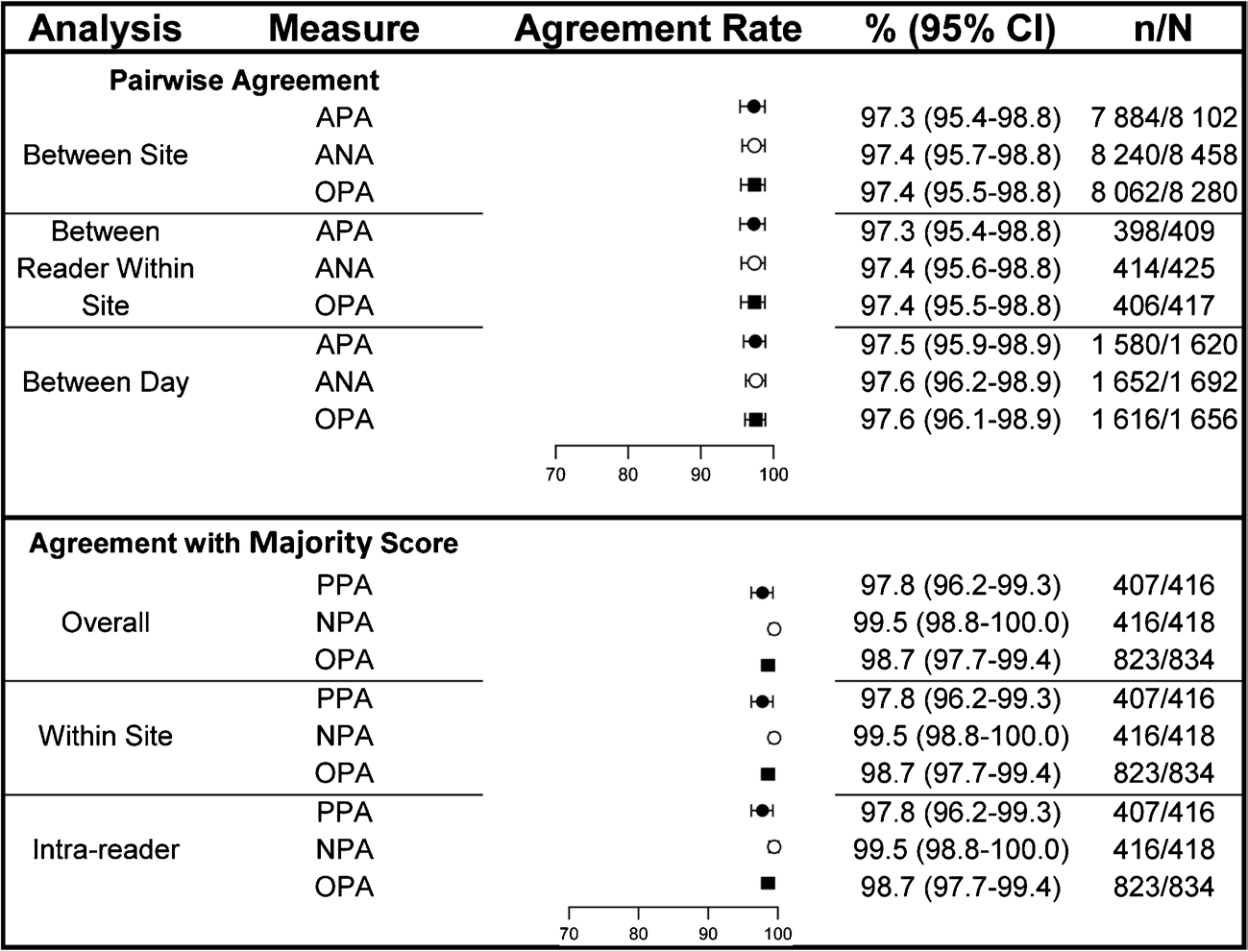
Interlaboratory reproducibility and pairwise reader agreements for HER2-low status (HER2-low or non-HER2-low). Slides included 28 breast carcinoma specimens (eleven IHC 0, seven IHC 1+, seven IHC 2+, three IHC 3+), including four borderline IHC 0/1+ cases. Slides were de-identified, randomized, and processed on the BenchMark ULTRA using the PATHWAY HER2 (4B5) antibody (and CONFIRM Negative Control Rabbit Ig) with the U PATHWAY HER2 (4B5) staining procedure. Two pathologists per 3 test sites read slides five times on different days. Two-sided 95% CIs were calculated using the percentile bootstrap method from 2000 bootstrap replicates. The majority score (or score most frequently assigned from multiple independent readers) was used for analyses. ANA, average negative agreement; APA, average positive agreement; CI, confidence interval; HER2, human epidermal growth factor receptor 2; IHC, immunohistochemistry; NPA, negative percent agreement; OPA, overall percent agreement; PPA, positive percent agreement

Overall inter-laboratory precision (3 sites, 2 pathologists per site) for HER2-low IHC status was high (OPA: 98.7%, 95% CI 97.7%–99.4%; Fig. 3). Reproducibility of HER2-low IHC status between readers within site (OPA: 97.4%, 95% CI 95.5%–98.8%), between days (OPA: 97.6%, 95% CI 96.1%–98.9%), and between sites (OPA: 97.4%, 95% CI 95.5%–98.8%) were also high.

**Fig. 4:**
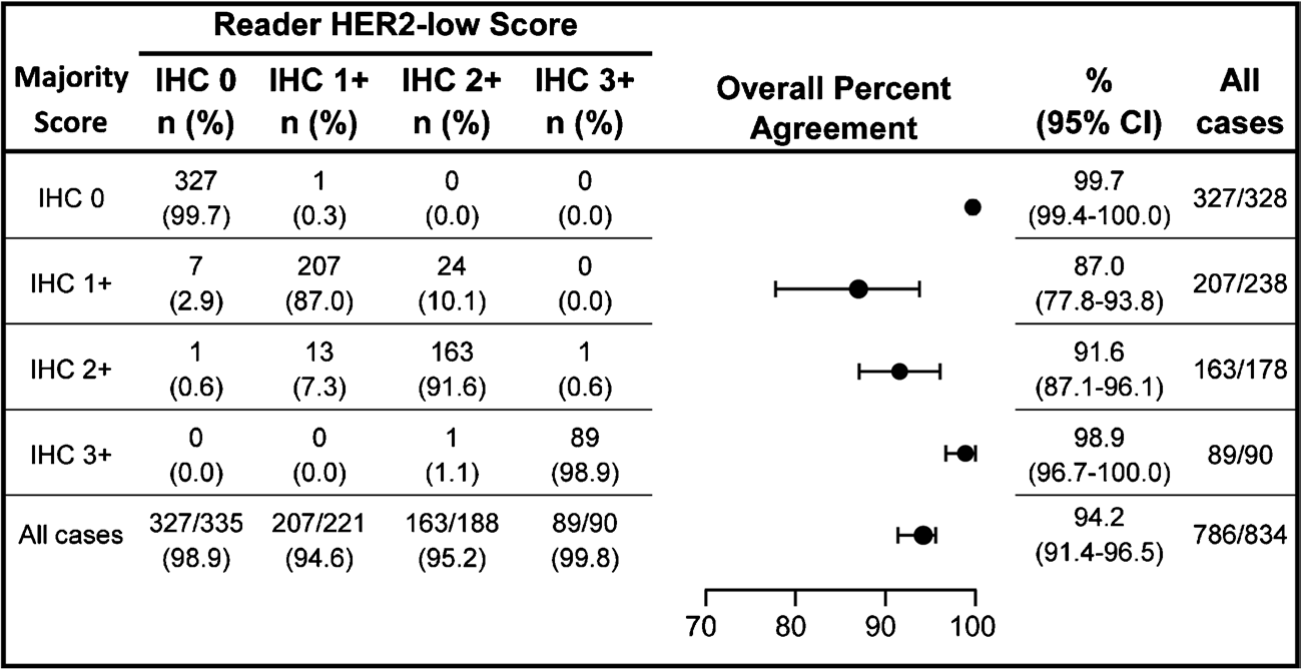
Agreement between interlaboratory reproducibility study readers and majority score for HER2-low IHC score. Slides included 28 breast carcinoma specimens (eleven IHC 0, seven IHC 1+, seven IHC 2+, three IHC 3+), including four borderline IHC 0/1+ cases. Slides were de-identified, randomized, and processed on the BenchMark ULTRA using the PATHWAY HER2 (4B5) antibody (and CONFIRM Negative Control Rabbit Ig) with the U PATHWAY HER2 (4B5) staining procedure. Two pathologists per three test sites read slides five times on different days. Two-sided 95% CIs were calculated using the percentile bootstrap method using 2000 bootstrap replicates. Majority scores (or highest frequency assigned score from multiple readers) was used for analyses. CI, confidence interval; HER2, human epidermal growth factor receptor 2; IHC, immunohistochemistry

The OPAs between individual reader HER2 IHC scores and the HER2 IHC majority score were higher for non-HER2-low specimens (IHC 0: 99.7%; IHC 3+: 98.9%) than HER2-low specimens (IHC 2+: 91.6%; IHC 1+: 87.0%), with most variability associated with slides scored IHC 1+ (Fig. 4). Most of the discordance observed at IHC 1+ was due to shift to the IHC 2+ category, which occurred for both individuals compared with consensus, and vice versa.

### HER2-low screening and sample characteristics in DB-04

The characteristics of samples stained with the PATHWAY HER2 (4B5) assay in DB-04 are presented in Supplementary Table 5. The distribution of tumor specimens was similar for screened and enrolled patients. Of the 1340 screened samples, 791 samples (59.0%) were from metastatic sites and 545 samples (40.7%) were from primary tumor, with 4 samples (0.3%) missing data. Out of 1340, 1183 (88.3%) were archival tissues (FFPE blocks or tissue sections). Nine hundred ninety-five out of 1340 (74.3%) were biopsy specimens, and 344 (25.7%) were excisions or resections. Six hundred seventy-nine (50.7%) of samples were collected in 2019 or thereafter, with only 111 (8.3%) collected prior to 2014. Out of 1340, 923 (68.9%) samples did not have associated data on historical testing methods, but when such information was provided, the prior local testing was performed mainly using the PATHWAY HER2 (4B5) or HercepTest (Dako Agilent Technologies) assays.

### Efficacy of T-DXd by sample type

**Fig. 5:**
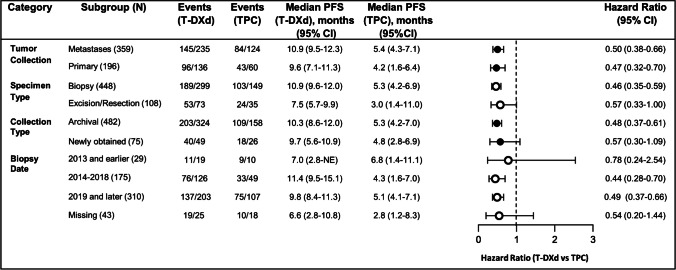
Progression-free survival of patients in DB-04 by sample type. CI, confidence interval; HER2, human epidermal growth factor receptor 2; HR, hazard ratio; NE, not evaluable; PFS, progression-free survival; TPC, therapy of physician’s choice

Among all the subjects randomized into DB-04, the median PFS was 9.9 months in the T-DXd group and 5.1 months in the physician’s choice group (HR for disease progression or death, 0.50; *P*<0.001) [13]. When median PFS was calculated according to the sample type used to establish HER2-low eligibility (i.e., metastasis vs primary; biopsy vs resection; archival vs fresh; date of collection), the benefit of T-DXd treatment was consistently observed across subgroups (Fig. 5). PFS HRs were within the range 0.442–0.573 for all subgroups, except for samples collected prior to 2014 (HR 0.783 [95% CI: 0.241–2.545]). However, the interpretation of this finding is difficult due to the small sample size of this subgroup (*n*=29 subjects total).

### Agreement between historic and central testing for HER2-low in DB-04

Historic and central testing results for DB-04 are provided in Supplementary Table 6, and a flow chart of sample screening is outlined in Supplementary Fig. 4. Of 1340 tumor samples submitted, 1108 samples had both prior historic and central HER2 testing results available. Overall, 823 out of 1060 (78%) of the samples that were designated as HER2-low (IHC 1+ or IHC 2+, ISH negative) by historic testing were found to be HER2-low via central testing with the PATHWAY HER2 (4B5) assay. Of the samples that were HER2-low by historic testing, 208 out of 1060 (19.6%) were found to be IHC 0 by central testing. Only 29 of 1060 (2.7%) historic HER2-low samples were determined to be IHC 2+/ISH+ or IHC 3+ by the central laboratory. This suggests that distinguishing between IHC 0 and IHC 1 may be more challenging than identification of HER2-positive tumors.

An analysis of factors that potentially contributed to discordance between historical test and central testing for HER2-low is provided in Supplementary Table 7. Concordance between historical and central laboratory test result was higher in North America (OPA 85.3%) and Asia (excluding China) (OPA 85.3%) than in Europe/Israel (OPA 69.8%) and China (OPA 67.6%). Concordance was lowest for samples collected before 2014 (OPA 64.0%), when compared with 2014–2018 (OPA 74.6%) or later (OPA 78.6%).

## Discussion

With the recent approval of T-DXd for HER2-low metastatic breast cancer in the USA, EU, and other major regions, unresectable metastatic breast cancer patients with tumors defined as IHC 1+ or IHC 2+ /ISH- now have a highly efficacious targeted treatment option after first line chemotherapy. Accurate testing of HER2 by IHC is critically important for this patient population. The PATHWAY HER2 (4B5) assay described in this report has also been recently approved by the FDA for the selection of patients for T-DXd treatment based upon the data presented herein, and multiple regional regulatory approvals are currently being sought, including in the EU under the new IVDR regulations. The PATHWAY HER2 (4B5) assay for HER2-low assessment is currently the only FDA-approved HER2 IHC Companion Diagnostic assay for assessing HER2-low expression.

In this study, we found strong inter- and intra-reader concordance between pathologists in both our precision and inter-laboratory reproducibility studies (Figs. 3 and 4). This contrasts with several recent studies [14, 15], in which concerns were expressed about the application of HER2 IHC for identifying HER2-low tumor expression. Specifically, inter-reader concordance for discriminating between IHC 0 and IHC 1+ was found to be poor, which led authors to speculate that patients could be improperly treated with T-DXd. There are several factors that could have contributed to the discordance between IHC 0 and HER2-low in these recent studies. First, the lack of clinical utility for the IHC 1+ category has historically not necessitated accurate distinction between IHC 0 and IHC 1+. In the Fernandez et al. study, pathologists were not told that assessing all HER2 expression levels (IHC 0–3+) was a study objective and admitted that if they had been informed that concordance at IHC 0 and IHC 1+ was going to be assessed, they would have put more effort into accurately evaluating these categories [14]. The inter- and intra-reader studies reported were performed by experienced Ventana pathologists, and so may be considered as best-case results. However, high concordance was also observed in the interlaboratory study, where testing was performed in routine clinical laboratories that is likely to more accurately reflect testing capabilities in the real world.

The 2023 update to the ASCO/CAP guidelines now include the clinical relevance of HER2-low [6]. Pathologists participating in DB-04 and in the analytical studies were trained on the HER2-low scoring algorithm with training materials that were developed such that tissue cases within the HER2-low category can be appropriately assessed. This likely enabled a high degree of HER2-low status agreement for these analyses. Furthermore, the interpretations in the current study were performed using glass slide and light microscopy, as opposed to utilizing the scanned images used in the Fernandez et al. study, which could have potentially affected HER2 evaluation at lower levels of expression. Consequently, the results of the current study may better reflect a real-world clinical setting where HER2-low is an actionable diagnosis.

The rate of disagreement between the prior local and the centrally determined HER2-low status was approximately 22%, with the majority of discordance occurring with samples scored as HER2 IHC 0 centrally. The differences observed are likely attributable to several key factors. First, prior to T-DXd, there was no clinical need to accurately distinguish patients with HER2-low tumors among those whose tumors did not overexpress HER2, as the therapeutic strategies were the same regardless, and therefore there was no prior need to accurately differentiate IHC 0 from IHC 1+. Also, there were no restrictions in DB-04 regarding the methodology and scoring guidelines used for the historical HER2 testing. As an example, most HER2 scoring guidelines prior to the 2013 ASCO/CAP update did not require a minimum percentage of stained tumor cells for assigning an IHC 1+ score. Our observation that disagreement between prior local and central HER2-low status in DB-04 was higher for samples collected in/prior to 2013 could be due to these differences in scoring guidelines. Also, loss of tissue antigenicity for older samples cannot be ruled out. For patients whose tumor HER2 IHC status was derived prior to 2014 or using a different assay, careful considerations should be made for whether HER2-low status should be re-confirmed using the PATHWAY HER2 (4B5) assay on a freshly-cut section.

Based on experience with HER2 testing following trastuzumab approval, where local versus central concordance for HER2-positivity scoring showed marked improvements over time (initial discordance for HER2 status of 52.4% was reduced to 8.4% in later studies), we anticipate improvements in HER2-low scoring as more pathologists receive training and education for HER2-low, and HER2-low assessment becomes standard routine clinical practice and is included in guidelines. Consistent with this, a recent study found improvements in HER2-low scoring after training [16].

An additional consideration in HER2 IHC assessment is the lack of assay standardization. We found that protocol deviations can cause staining and scoring variability, particularly within the IHC 0 and IHC 1+ samples. However, when using the recommended staining protocol agreements in HER2-low status were high across all precision, repeatability and inter-laboratory reproducibility studies. Notably, according to a review of a recent external quality assurance (EQA), of the 248 participating laboratories using the PATHWAY HER2 (4B5) assay, only 50 used the recommended staining procedure for the assay [17]. Based on our findings and the data from EQA groups, deviations in staining protocols may in part explain why we found discordance between prior local and centralized testing in DB-04. In addition, we found that adherence to standard ASCO/CAP recommended preanalytical procedures (tissue section thickness, fixation, etc.) were also critical to ensure accurate assessment of HER2-low status.

Despite the high level of agreement for these analytical studies, some discrepancies between readers did occur. The majority of inter-reader discordance was with slides scored as IHC 1+ by majority reference score; 87% of individual pathologist scores were concordant with majority score, with 10.1% scored as IHC 2+ (which would not affect the eligibility of the respective patient for T-DXd treatment in the metastatic breast cancer setting) and 2.9% were scored as IHC 0 (a change in HER2-low status and therefore, potentially, in treatment decisions). For IHC 2+, 15/178 (8.4%) of results showed discordance with majority score, with only 1 sample being scored as IHC 0 and thus the patient potentially being deemed ineligible for T-DXd treatment. This level of inter-reader discordance is acceptable in clinical practice.

Patients whose tumors are HER2-low may have higher heterogeneity of HER2 expression, which has led to some concern that smaller biopsy samples may not reflect overall HER2 expression across the tumor [18]. The samples used for HER2-low analysis in DB-04 reflected the types generally observed in metastatic breast cancer and included both primary and metastatic samples. The majority were archival specimens, with approximately 80% being biopsy samples. Efficacy of T-DXd in DB-04 was consistent, regardless of sample type used for HER2-low determination. Of particular note, efficacy remained high in patients with HER2-low breast cancers diagnosed based on a biopsy sample. Several studies have shown good concordance for HER2-positive determination by IHC between biopsies and resections [5, 19], and biopsies are widely deemed suitable for HER2 status testing. Our results demonstrate that biopsies are also suitable for HER2-low assessment.

At this time, a number of commercially available, regulatory authority-approved HER2 IHC testing kits are utilized in clinical practice to help identify patients with HER2-positive tumors who may be suitable for treatment with HER2-targeted therapies. The PATHWAY HER2 (4B5) assay is currently the only testing methodology validated for the unresectable or metastatic HER2-low breast cancer indication. Whether HER2 IHC assays other than PATHWAY HER2 (4B5) can be used to identify subsets of breast cancer cases with lower levels of HER2 expression, who are candidates for treatment with T-DXd, requires further investigation, although studies have found HER2-low prevalence to be similar, regardless of assay used [20–22]. In cases where a patient has multiple HER2 results from different assays, the PATHWAY HER2 (4B5) result should be given most weight when considering if patients are eligible for T-DXd in the HER2-low breast cancer setting, until additional data on the clinical utility of other assays for HER2-low scoring becomes available. Other technologies may offer more quantitative assessments of HER2 levels in tumors; however, their clinical utility has not been established, and their availability for routine assessment in clinical practice is typically very limited. DB-04 demonstrated that T-DXd was effective in patients whose tumors are HER2 IHC 1+ or IHC 2+ but did not assess efficacy in patients with lower HER2 IHC expression. The threshold of HER2 expression that predicts HER2-targeted ADC therapy efficacy remains unknown.

In summary, the analytical study results presented herein demonstrate that the PATHWAY HER2 (4B5) assay is highly precise and reproducible for determining HER2-low status when IHC samples are processed in a laboratory following the assay recommendations and scored by trained pathologists following ASCO/CAP guidelines. The use of this analytically validated assay in DB-04 demonstrated T-DXd efficacy improvements over chemotherapy of physician’s choice were observed regardless of sample type used for determining HER2-low status.

### Supplementary Information

Below is the link to the electronic supplementary material.

Supplementary file1 (PDF 907 KB)

## Data Availability

Anonymized individual participant data (IPD) and applicable supporting clinical trial documents may be available upon request at (https://vivli.org). In cases where clinical trial data and supporting documents are provided pursuant to our company policies and procedures, Daiichi Sankyo Companies will continue to protect the privacy of the company and our clinical study patients. Details on data sharing criteria and the procedure for requesting access can be found at this web address: https://vivli.org/ourmember/daiichi-sankyo.
