# Supplementary material for: Analytical and clinical validation of PATHWAY Anti-HER-2/neu (4B5) antibody to assess HER2-low status for trastuzumab deruxtecan treatment in breast cancer
Source: Virchows Arch. 2023 Oct 20;484(6):1005–14. doi: 10.1007/s00428-023-03671-x (PMC11186906; doi:10.1007/s00428-023-03671-x)
Supplement: Supplementary file 1 — Supplementary file1 (PDF 907 KB) [file 428_2023_3671_MOESM1_ESM.pdf]

Analytical and Clinical Validation of PATHWAY Anti-HER-2/neu (4B5) Antibody to Assess HER2-low Status for Trastuzumab Deruxtecan Treatment in Breast Cancer, Supplemental Material

Charo Garrido, Melissa Manoogian, Dhiraj Ghambire, Shawn Lucas, Maha Karnoub, Matthew T. Olson, David G. Hicks, Gary Tozbikian, Aleix Prat, Naoto T. Ueno, Shanu Modi, Wenqin Feng, Judith Pugh, Ching Hsu, Junji Tsurutani, David Cameron, Nadia Harbeck, Qijun Fang, Shirin Khambata-Ford, Xuemin Liu, Landon J. Inge, Patrik Vitazka

* Corresponding authors; Charo Garrido, Daiichi Sankyo Inc., Basking Ridge, NJ (rgarrido@dsi.com) and Melissa Manoogian, Roche Tissue Diagnostics, Tuscon, AZ (melissa.manoogian@roche.com)

Supplemental Methods

Tissue Specimens for Preanalytical, Staining, and Analytical Validation Studies

Formalin-fixed paraffin-embedded (FFPE) breast cancer tumor specimens sourced from commercial vendors were used in analytical studies, except where indicated. All specimens were provided de-identified. Cases for analytical study inclusion were chosen based upon a HER2 IHC score assigned through pre-screening at Roche Tissue Diagnostics by a single pathologist or by consensus score (score assigned by two pathologists, plus a third pathologist when there were disagreements).

Preanalytical Tissue Processing Studies

The human breast cancer cell line, MDA-MB-361, which has demonstrated IHC 2+ HER2 expression [1], was used to generate xenograft tumors. To evaluate the impact of ischemia, xenograft tumors were harvested, kept at room temperature without solution until their respective time-point, and then fixed in 10% neutral-buffered formalin (NBF) for 24 hours before processing. The MDA-MB-361 xenograft tumors were sectioned, stained, and scored as indicated. For studies assessing different fixatives, xenograft tumors were harvested and placed into the indicated fixatives for 24 hours before processing. For tissue section thickness studies, FFPE blocks were sectioned from 2 to 10µM and stained as described. For cut slide stability, sections were stored at the indicated temperatures and humidity levels and stained at pre-determined time points to determine the impact of these parameters upon staining performance.

Immunohistochemistry

FFPE blocks were sectioned at 4 µm and mounted onto positively charged glass slides. Except where noted, all slides were stored at 4°C in low relative humidity and stained within

four months. All slides were stained on the VENTANA BenchMark ULTRA staining instrument (VENTANA Medical Systems, Inc.) equipped with VENTANA VSS Version 12.3 software. IHC staining was performed using the current FDA-approved, CE-marked recommended staining procedure for anti-HER2 IHC analysis (Supplemental Table 1), with the following staining protocol steps:

1. Deparaffinization
2. Antigen unmasking (instrument setting Cell Conditioning 1: mild/36 minutes at 100°C)
3. Antibody incubation with the PATHWAY anti-HER2/neu (4B5) Rabbit Monoclonal Antibody) (also referred to as PATHWAY HER2 (4B5) assay) or VENTANA CONFIRM rabbit negative control Ig (also referred to as negative reagent control [NRC]) (instrument setting 12 minutes at 36°C)
4. Staining with VENTANA *ultra*View Universal DAB IHC Detection Kit
5. Counterstaining with hematoxylin II (4 minutes) and then bluing reagent (4 minutes)

The PATHWAY HER2 4-in-1 Control Slide stained with PATHWAY HER2 (4B5) antibody was used to ensure appropriate HER2 staining on each instrument run. FFPE tumor specimen-matched tissue specimens stained with the NRC were also reviewed for the presence and acceptability of nonspecific background staining. Once control slides were deemed acceptable, the PATHWAY HER2 (4B5) antibody-stained test slides were evaluated.

Staining Study with Variable Cell Conditioning and Antibody Incubation Times

Variances in the IHC staining protocol, particularly in the incubation time of the cell conditioning and antibody incubation steps, can significantly impact staining intensity [2]. To evaluate whether IHC score and/or HER2-low status is affected by using alternative assay

staining protocols, 13 FFPE resections (including 11 borderline cases near the 10% cutoff between IHC 0 and 1+) were selected and stained using the recommended staining protocol versus alternative protocols with variations in the cell conditioning and/or antibody incubation staining steps. The recommended incubation times for cell conditioning and antibody incubation are 36 minutes and 12 minutes, respectively. Reference slides from each FFPE tumor specimen were created using the recommended staining protocol with the PATHWAY HER2 (4B5) antibody and NRC. Experimental slides for each specimen were generated using the alternative staining protocols with the PATHWAY HER2 (4B5) antibody and NRC. Slides were randomized, and stained sections were evaluated using the HER2 IHC scoring method described in the main Methods section. All slides (reference and experimental) were evaluated by pathologists blinded to the reference score and staining procedure.

Precision and Reproducibility Studies

Intra- and Inter-Reader Precision

For inclusion in the reader precision study, a consensus score for HER2-low status was established for each case by a separate group of two pathologists. The precision study encompassed assessments of inter-reader and intra-reader concordance. This study included 100 breast carcinoma specimens (83 resections and 17 core needle biopsies) spanning the range of HER2 expression (41 IHC 0, 25 IHC 1+, 25 IHC 2+, and 9 IHC 3+), including 11 borderline specimens (10 borderline specimens near the 10% cutoff between IHC 0 and 1+ and 1 between IHC 2+ and IHC 3+). These samples were distinct from those used in other analytical studies. Slides for each specimen were stained with the BenchMark ULTRA staining instrument using the PATHWAY HER2 (4B5) antibody (or NRC) and the current FDA, CE-marked recommended staining procedure. Slides were de-identified, randomized, and evaluated twice by three internal (Roche) pathologists, to give six complete data sets,

with a minimum of a two-week washout period between the two reads. All Roche pathologists had significant experience in evaluating the HER2 4b5 assay in breast (>5 years).

For inter-reader precision, the HER2-low status determined by one pathologist was compared to that determined by another pathologist for each case and deemed concordant or discordant. For the purposes of statistical analysis, cases with IHC 1+ or 2+ were classified as HER2-low and cases with IHC scores of 0 and 3+ were classified as non-HER2-low. Average negative agreement (ANA) and average positive agreement (APA) were calculated for each pair of readers and aggregated across reader pairs to determine the overall APA and ANA. For intra-reader precision, the HER2-low status (HER-2-low or non-HER2-low) from each pathologist's first read of a case was compared with that from their second read of the case and deemed concordant or discordant. The APA and ANA were calculated for each reader and aggregated across readers to determine the overall APA and ANA. For APA and ANA in each pairwise comparison, two-sided 95% confidence intervals (CIs) were calculated using the transformed Wilson score method. For APA and ANA aggregated across pairwise comparisons, two-sided 95% CIs were calculated using the percentile bootstrap method.

Intermediate Precision and Repeatability

Intermediate precision and repeatability studies were performed between three PATHWAY HER2 (4B5) antibody reagent lots, between three *ultra*View Universal DAB detection kit lots, between three BenchMark ULTRA staining instruments, between three days, and within two different instrument runs. For the antibody lot, detection kit lot and instrument studies, replicate slides were sectioned from 24 unique breast carcinoma tissue specimens and used for staining and evaluation. For between-day precision and within-instrument run repeatability studies, replicate slides were prepared from the same 24 unique breast carcinoma tissue specimens, stained with one antibody reagent and detection kit lot, and run

on one instrument across three non-consecutive days. Of the 24 specimens (six IHC 0, six IHC 1+, six IHC 2+, and six IHC 3+) included in the study, four were HER2-low borderline cases (IHC 0/1+ at the 10% cutoff). Samples were randomized and de-identified, and one pathologist read all slides. For the purposes of statistical analysis, cases with IHC 1+ or 2+ were classified as HER2-low, and cases with IHC scores of 0 and 3+ were classified as non-HER2-low. Positive percent agreement (PPA), negative percent agreement (NPA), and overall percent agreement (OPA) for HER2-low status (HER2-low versus non-HER2-low) were evaluated. Two-sided 95% CIs were calculated from 2 000 bootstrap replicates using the percentile bootstrap method. For observations of 100.0%, the 95% CIs were calculated using the Wilson score method.

Interlaboratory Reproducibility

Interlaboratory reproducibility studies were performed at three external laboratories. In total, 28 breast cancer tissue specimens (eleven IHC 0, seven IHC 1+, seven IHC 2+, and three IHC 3+) were selected; of which four were IHC 0/1+ borderline cases. Sites were provided with five sets of slides to stain; laboratory personnel-stained slides on five nonconsecutive days spanning over ≥20 days, with the first and last staining days spaced ≥20 days apart. Slides were de-identified and randomized according to a randomization schedule; thus, slides were stained and read in differing orders. Slides were stained using the recommended PATHWAY HER2 (4B5) assay procedure. Two pathologists per site (i.e., six pathologists in total) read slides. For the purposes of statistical analysis, cases with IHC 1+ or 2+ were classified as HER2-low and cases with IHC scores of 0 and 3+ were classified as non-HER2-low. The APA, ANA, PPA, NPA, and OPA for HER2 IHC scores and/or HER2-low statuses were evaluated. Two-sided 95% CIs were calculated using the percentile bootstrap method using 2 000 bootstrap replicates. For observations of 100.0%, the 95% CIs were calculated using the Wilson score method (for PPA, NPA, and OPA) or the transformed Wilson score

method (APA and ANA) [3]. For the interlab reproducibility study, each site had two pathologist readers. All readers are board certified anatomic/surgical pathologists with an average of 13 years of clinical practice (range 6-23 years). 1 of 6 readers was a specialist in breast pathology.

1. Boyer JZ, Phillips GDL, Nitta H, et al (2021) Activity of trastuzumab emtansine (T-DM1) in 3D cell culture. Breast Cancer Res Treat 188:65–75. https://doi.org/10.1007/s10549-021-06272-x
2. Taylor CR, Levenson RM (2006) Quantification of immunohistochemistry—issues concerning methods, utility and semiquantitative assessment II. Histopathology 49:411–424. https://doi.org/https://doi.org/10.1111/j.1365-2559.2006.02513.x
3. Yee L (2016) Immunohistochemistry devices: Analytical and clinical validation. In: Presented at 9th FDA/AdvaMed Medical Devices and Diagnostics Statistical Issues Conference

Supplemental Figures and Tables

Supplemental Table 1: Recommended staining protocol for determining HER2-low breast cancer status using the BenchMark ULTRA platform and PATHWAY HER2 (4B5) assay

Procedure/Step	Recommended Protocol
Deparaffinization	Selected
Cell Conditioning<br>(Antigen Unmasking)	Mild (36 minutes, 100°C) Cell Conditioning 1
PATHWAY HER2 (4B5) rabbit monoclonal primary antibody or CONFIRM rabbit negative control Ig	12 minutes, 36°C
Counterstain	Hematoxylin II, 4 minutes
Post Counterstain	Bluing Reagent, 4 minutes

DAB: 3,3’-diaminobenzidine; HER2: human epidermal growth factor receptor 2.

Supplemental Table 2: Failures observed when varying antibody and cell conditioning incubation times in the PATHWAY HER2 (4B5) assay for HER2-low assessment

		Reference IHC: 1+ n=4			Reference IHC: 2+ n=3		Reference IHC: 3+ n=3		Observed Failure	
Cell Conditioning Incubation Time (min)	Antibody Incubation Time (min)	Test IHC: 0	Test IHC: 1+	Test IHC: 2+	Test IHC: 1+	Test IHC: 2+	Test IHC: 2+	Test IHC: 3+	HER2-low Status Change/ Potential Change	Negative Control Staining Unacceptable
20	12	0	4	0	1	2	2	1	yes	no
20	16	2	2	0	1	2	1	2	yes	no
20	20	2	2	0	0	3	0	3	yes	yes
20	24	1	3	0	0	3	0	3	yes	yes
20	28	0	4	0	0	3	0	3	no	yes
36	20	0	4	0	0	3	0	3	no	no
36	28	0	4	0	0	3	0	3	no	yes
36	32	0	3	1	0	3	0	3	yes	yes
36	36	0	4	0	0	3	0	3	no	yes
52	12	0	4	0	0	3	0	3	no	no
52	16	0	3	1	0	3	0	3	yes	no
52	28	0	3	1	0	3	0	3	yes	no
52	32	0	2	2	0	3	0	3	yes	yes
52	36	0	3	0	0	4	0	3	no	yes
64	12	0	3	0	0	4	0	3	no	no
64	16	0	3	0	0	4	0	3	no	no
64	24	0	3	0	0	4	0	3	no	no
64	32	0	2	1	0	4	0	3	no	no
64	36	0	3	0	0	4	0	3	no	yes

HER2: human epidermal growth factor receptor 2; IHC: immunohistochemistry.Test samples were processed on a BenchMark ULTRA using the PATHWAY HER2 (4B5) antibody with the U PATHWAY HER2 (4B5) staining procedure, with the indicated adjustments in the cell conditioning and antibody incubation times. Samples were compared with a sample processed using the recommended PATHWAY HER2 (4B5) assay of cell conditioning for 36 minutes and antibody incubation time of 12 minutes (reference IHC sample). Cases that switch between IHC 1+ and IHC 2+ are designated as having potential change in HER2-low status (which would depend on the ISH status of the sample).

Supplemental Figure 1: DB-04 study design and HER2-low screening strategy

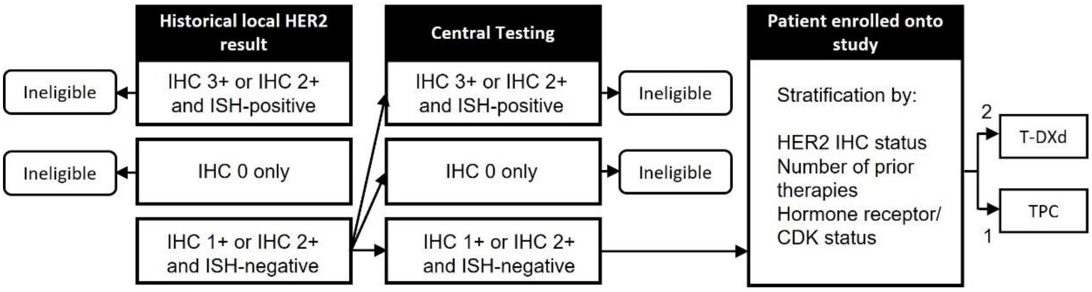

Patients recruited onto DB-04 were required to have HER2-low tumors by local/historical test. HER2-low status was then confirmed by central testing with the PATHWAY HER2 (4B5) assay and, where appropriate, the VENTANA INFORM HER2 dual ISH DNA probe cocktail assay. Only patients with samples confirmed as HER2-low by central test were randomized. DB-04: DESTINY-Breast04; HER2: human epidermal growth factor receptor 2; IHC: immunohistochemistry; ISH: in situ hybridization; TPC: treatment of physician's choice; T-DXd: Trastuzumab deruxtecan.

Supplemental Figure 2: Comparison of fixatives for use in breast cancer xenograft tumor staining with the PATHWAY HER2 (4B5) assay optimized for HER2-low scoring

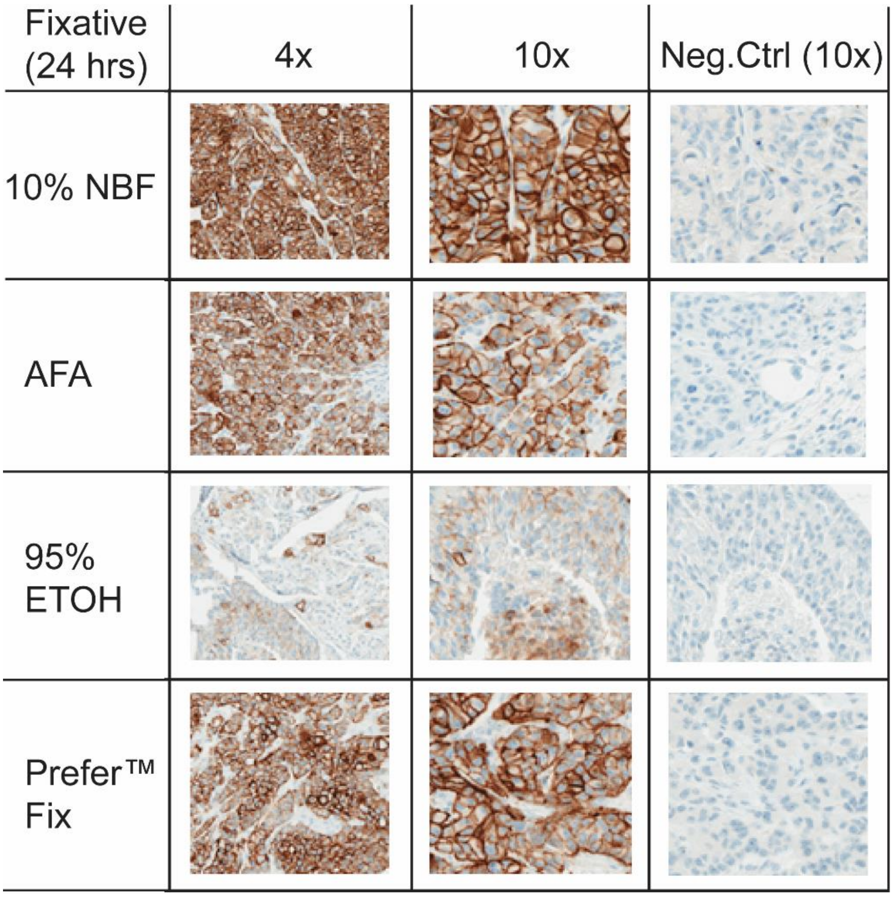

Compared with 10% NBF, use of AFA, 95% alcohol, and Prefer™ fixatives resulted in decreased PATHWAY HER2 (4B5) assay staining. AFA: alcohol formalin acetic acid; HER2: human epidermal growth factor receptor 2; NBF: neutral buffered formalin.

Supplemental Table 3. Agreement in HER2 IHC scores with a 4 µm reference for breast cancer tissues of different section thicknesses

Test Thickness	Agreement with 4µm Reference n/N (%)
2µm	12/12 (100%)
3µm	12/12 (100%)
4µm	12/12 (100%)
5µm	12/12 (100%)
6µm	12/12 (100%)
7µm	10/12 (83%)

HER2: human epidermal growth factor receptor 2; IHC: immunohistochemistry.Samples were processed in duplicate with 6 formalin-fixed paraffin-embedded breast carcinoma tissue cases: three IHC 0 (including one borderline case), one IHC 1+, two IHC 2+, and one IHC 3+.

Supplemental Figure 3: Cut slide stability of breast cancer tissue samples stained with PATHWAY HER2 (4B5) assay and scored for HER2-low status at two different temperatures and RHs, day 0 to month 9

Day 0 Staining	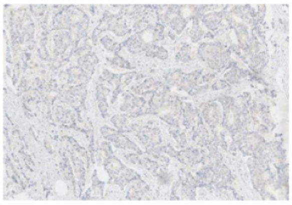			
	Storage Temperature and Humidity			
Time Point	5°C±3°C<br>RH 15%±10%	5°C±3°C<br>RH 85%±10%	30°C±5°C<br>RH 15%±10%	30°C±5°C<br>RH 85%±10%
Day 7	12/12 (100%)	8/8 (100%)	8/8 (100%)	6/12 (50%)
Day 45	12/12 (100%)	8/8 (100%)	8/8 (100%)	4/12 (33%)
Month 2	12/12 (100%)	8/8 (100%)	8/8 (100%)	2/12 (17%)
Month 3	12/12 (100%)	8/8 (100%)	8/8 (100%)	2/8 (25%)
Month 4	12/12 (100%)	8/8 (100%)	8/8 (100%)	2/6 (33%)
Month 6	12/12 (100%)	6/8 (75%)	2/8 (25%)	2/6 (25%)
Month 7	8/12 (67%)	4/8 (50%)	2/8 (25%)	2/4 (50%)
Month 9	6/12 (50%)	Not assessed	Not assessed	Not assessed

Cell values indicate number of slides in agreement with day 0 HER2-low IHC score/N (overall percent agreement). Sample types included tissue resections, needle core biopsies, and mock needle cores, and the control sample used was the PATHWAY HER2 4-in-1 control slide. Slides were stained on the BenchMark ULTRA using the PATHWAY HER2 (4B5) assay (and VENTANA CONFIRM rabbit negative control Ig) and the U PATHWAY HER2 (4B5) staining procedure. 100% agreement was considered acceptable/passing score. HER2: human epidermal growth factor receptor 2; IHC: immunohistochemistry; RH: relative humidity.

Supplemental Table 4: Intra-reader and Inter-reader precision for HER2-low IHC Status using the PATHWAY HER2 (4B5) assay on the BenchMark ULTRA

Analysis	Type	n/N	Percent Agreement (95% CI)
Intra-reader Precision	APA	312/333	93.7 (90.9-96.4)
	ANA	246/267	92.1 (88.0-95.6)
	OPA	279/300	93.0 (90.0-96.0)
Inter-reader Precision	APA	300/332	90.4 (85.8-94.3)
	ANA	236/268	88.1 (82.1-93.0)
	OPA	268/300	89.3 (84.7-94.0)

ANA: average negative agreement; APA: average positive agreement; CI: confidence interval; HER2: human epidermal growth factor receptor 2; OPA: overall percent agreement.

In total, 100 breast cancer specimens (83 resections and 17 needle core biopsies) spanning the range of HER2 expression, including 11 borderline HER2-low positive/negative cases, were stained and read twice by three pathologists. A washout period of two weeks occurred between the two reads. Two-sided 95% CIs were calculated using the percentile bootstrap method from 2000 bootstrap replicates.

Supplemental Table 5: Sample Characteristics of Patients Screened and Enrolled into DB-04

	All Screened (N=1340)		Enrolled (N=557)	
**Feature**	**N**	**%**	**N**	**%**
**Tumor Location**				
Primary	545	40.7	196	35.2
Metastases	791	59.0	359	64.4
Missing	4	0.3	2	0.4
**Specimen Type**				
Biopsy	995	74.3	448	80.4
Excision/Resection	344	25.7	108	19.4
Missing	1	0.1[a]	1	0.2
**Collection Type**				
Archival Tissue	1 183	88.3	482	86.5
Wet (Fresh) Tissue	157	11.7	75	13.5
**Specimen Collection Date**				
2013 or earlier	111	8.3	29	5.2
2014-2018	483	36.0	175	31.4
2019 to present	679	50.7	310	55.7
Missing	67	5.0	43	7.7
**Historical HER2 Test Type**				
PATHWAY HER2 (4B5) assay	260	19.4	106	19.0
HerceptTest	131	9.8	59	10.6
Assays using other antibodies (CB11 [VENTANA], EP3, SP3 clone) or ISH	26	1.9	9	1.6
Missing	923	68.9	383	68.8

HER2: human epidermal growth factor receptor 2; IHC: immunohistochemistry; ISH: in situ hybridization.
“Fresh” tissue refers to the taking of a new biopsy, which was processed to FFPE sections for IHC analysis.
[a]Total does not add up to 100% because of rounding.

Supplemental Figure 4: Sample Flow Chart for HER2-low Testing Results in DB-04

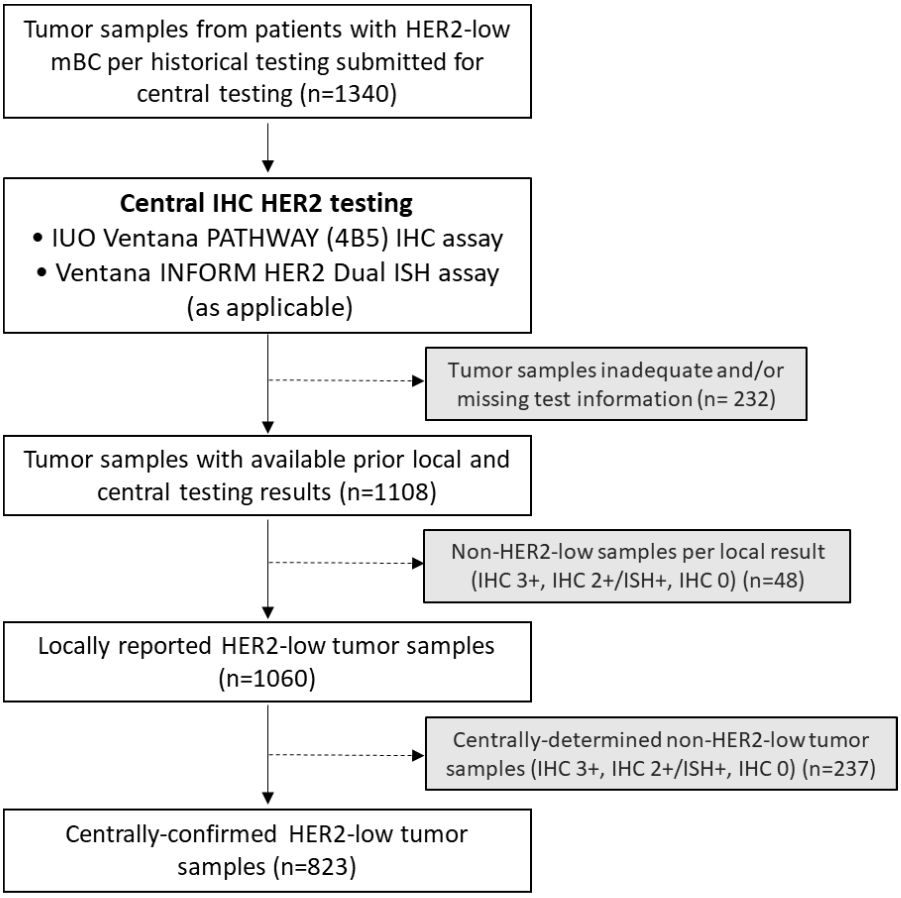

mBC: metastatic breast cancer; IUO: investigational use only; IHC: immunohistochemistry; ISH: in situ hybridization

Supplemental Table 6: Central versus Historic HER2 scores in DB-04

		Prior Local HER2 Score				All
		IHC 0	IHC 1+	IHC 2+/ ISH negative	IHC 2+/ ISH positive	
Central HER2 Score	IHC 0	18	157	51	2	228
	IHC 1+	18	344	126	3	491
	IHC 2+/ ISH negative	5	122	231	.	358
	IHC 2+/ ISH positive	.	9	11	1	21
	IHC 3+	1	2	7	.	10
	All	42	634	426	6	1108

DB-04: DESTINY-Breast04; HER2: human epidermal growth factor receptor 2; IHC: immunohistochemistry; ISH: in situ hybridization.

Supplementary Table 7: Concordance Between Historic and Central HER2-low Scoring by Region and Sample Collection Date

Feature	N (%)	OPA (95% CI)
**Region of Origin**		
North America	252 (22.7)	85.3 (80.9-89.7)
Europe & Israel	461 (41.6)	69.8 (65.7-74.0)
Asia (excluding China)	287 (25.9)	81.8 (77.4-86.3)
China	108 (9.7)	67.6 (58.8-76.3)
**Specimen Collection Date**		
2013 or earlier	94 (8.5)	63.8 (54.1-73.5)
2014-2018	421 (38.0)	74.8 (70.7-79)
2019 to present	555 (50.1)	78.6 (75.1-82.0)
Missing	38 (3.4)	89.5 (79.7-99.2)

Concordance was assessed for categorization of samples as HER2-low versus non-HER2-low. CI: confidence interval; HER2: human epidermal growth factor 2; OPA: overall percent agreement.
